# Human Chondrocytes from Human Adipose Tissue-Derived Mesenchymal Stem Cells Seeded on a Dermal-Derived Collagen Matrix Sheet: Our Preliminary Results for a Ready to Go Biotechnological Cartilage Graft in Clinical Practice

**DOI:** 10.1155/2021/6664697

**Published:** 2021-02-23

**Authors:** Quan Tran Dang, Thao Duy Huynh, Francesco Inchingolo, Gianna Dipalma, Alessio Danilo Inchingolo, Stefania Cantore, Gregorio Paduanelli, Kieu Cao Diem Nguyen, Andrea Ballini, Ciro Gargiulo Isacco, Cong Toai Tran

**Affiliations:** ^1^University of Medicine and Pharmacy, Ho Chi Minh City, Vietnam; ^2^Pham Ngoc Thach University of Medicine, Ho Chi Minh City, Vietnam; ^3^Department of Interdisciplinary Medicine, University of Bari “Aldo Moro”, Bari, Italy; ^4^Department of Biosciences, Biotechnologies and Biopharmaceutics, Campus Universitario “Ernesto Quagliariello”, University of Bari “Aldo Moro”, Bari, Italy; ^5^Department of Precision Medicine, University of Campania “Luigi Vanvitelli”, Naples, Italy; ^6^School of Medicine, Viet Nam National University, Ho Chi Minh City, Vietnam

## Abstract

**Background:**

The articular cartilage is unique in that it contains only a single type of cell and shows poor ability for spontaneous healing. Cartilage tissue engineering which uses mesenchymal stem cells (MSCs) and adipose tissue-derived mesenchymal stem cells (AT-MSCs) is considered an attractive treatment for cartilage lesions and osteoarthritis. The establishment of cartilage regenerative medicine is an important clinical issue, but the search for cell sources able to restore cartilage integrity proves to be challenging. The aim of this study was to create cartilage grafts from the combination of AT-MSCs and collagen substrates.

**Methods:**

Mesenchymal stem cells were obtained from human donors' adipose tissue, and collagen scaffold, obtained from human skin and cleaned from blood vessels, adipose tissues, and debris, which only preserve dermis and epidermis, were seeded and cultured on collagen substrates and differentiated to chondrocytes. The obtained chondrocyte extracellular matrix of cartilage was then evaluated for the expression of chondrocyte-/cartilage-specific markers, the Cartilage Oligomeric Matrix Protein (COMP), collagen X, alpha-1 polypeptide (COL10A1), and the Collagen II, Human Tagged ORF Clone (COL2A1) by using the reverse transcription polymerase chain reaction (RT-PCR).

**Results:**

Our findings have shown that the dermal collagen may exert important effects on the quality of in vitro expanded chondrocytes, leading in this way that the influence of collagen skin matrix helps to produce highly active and functional chondrocytes for long-term cartilage tissue regeneration.

**Conclusion:**

This research opens up the possibility of generating cartilage grafts with the precise purpose of improving the existing limitation in current clinical procedures.

## 1. Introduction

Cartilage is one of the most important structural tissues and together with bone is subjected to deterioration under the effect of multiple conditions such as aging, metabolic disorders, hormonal deficit, and traumatic injuries [1]. Compared to bone tissue, cartilage has poor or no capability of self-repairing due to a complete avascularity and lack of nerve and lymphatic support [1]. For this reason, therapeutic regenerative approaches have encountered strong limitations and poor outcomes [2]. The chondrocytes are cells of mesenchymal origin and form the 5% of the entire cartilage tissue though they work as main homeostasis factors producing and regulating the remaining 95% of cartilage compound made of extracellular matrix (ECM), of which collagen type 2 and proteoglycans (PGs) are the main components [3, 4].

**Figure 1:**
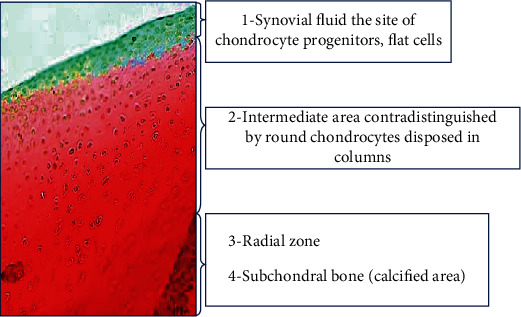
Structure of human cartilage. The cartilage tissue consists of 4 different substrates: 1, the superficial area, a mélange of synovial fluid, chondrocytes, stem cells, and flat cells; 2, an intermediate zone; 3, the radial zone; and 4, the subchondral calcified area close to the bone.

Cartilage tissue histologically consists of four substrates, the most superficial one in relation with the synovial fluid and chondrocyte progenitors, the intermediate area contradistinguished by round chondrocytes followed by the radial zone, and a final calcified area close to subchondral bone (Figure 1) [4, 5]. Any trauma leading to cartilage degeneration, arthritis, affects 26% of adult population of industrialized countries and constitutes one of the highest costs sustained by the national health system [3].

One of the great challenges in modern surgery and reconstructive medicine is to achieve an acceptable long-lasting result of joint biomechanical bone interface functionality and regeneration due to complexity of the inner structure mainly composed by cartilage and its extracellular matrix [5–7]. Despite the use of mesenchymal stem cells (MSCs), their secretome and interactions with bioscaffolds/biomaterials demonstrated to be a useful tool in regenerative medicine, showing significant differentiation capacity in endochondral bone formation; the chondrocyte hypertrophic end stage is almost inevitable [8, 9].

Currently, few approaches have been used such as surgical techniques like the use of autologous/allogeneic or cell regenerative approaches with the infiltration of autologous/allogeneic MSCs and chondrocytes [7, 10–13]. Limitations to these techniques are seen on medium long-term postsurgery/transplantation due to chondrocyte limited self-regenerative capacity and low number of transplanted cells during the implantation procedure; additionally, transplanted chondrocytes tend to lose their capacity of producing new ECM over the time, an event which contributes to generate either inflammation or infection with a consequent loss and/or rejection of both transplanted tissue and cells [14].

The aim of this study was to present an innovative procedure for the generation of a cartilage-like tissue by using a regenerative approach with adipose tissue-derived MSCs (AT-MSCs) seeded on human dermal collagen matrix obtained from human skin.

## 2. Materials and Methods

### 2.1. MSCs from Adipose Tissue Isolating Procedure

All procedures were performed under informed consent, in accordance with human study protocols approved by the Declaration of Helsinki for the reuse of human biospecimens in scientific research and the Vietnamese National Health Institute Committee of Biosolution (Research n93IRB-VN01013; grant number B2019-44-01). Fat tissues were originally collected from consented donors and tested for different panels of infectious agents such as HIV, HBV, HCV, and VDRL. Abdominal fat was harvested endoscopically in a lipoaspirate form and transferred to a laboratory facility for further process [15]. Fat was collected in proper sterile containers with DMEM/F12 (Gibco, Grand Island NY, USA), FBS 10%, (Gibco, Grand Island NY, USA), and gentamicin 50 *μ*g/ml (Gibco, Grand Island NY, USA). Tissue was then transferred into dishes and cleaned with a PBS-streptomycin-penicillin solution, foreign tissues were removed, and fat tissues were finely chopped and transferred into a dispase-collagenase enzymatic solution (3 : 1 ratio) and incubated for 90 minutes at 37°C. The sample was then centrifuged (Universal 32, Zentrifugen, Germany) for 5 minutes at a speed of 3,000 rpm; the bottom sediment was collected and filtered by a cell strainer with diameter of 70 *μ*M (BD Falcon™). The obtained material was recentrifuged at a speed of 3,000 rpm for 5 minutes, and the sediment was collected and incubated into T-25 cm^2^ flask (Nunc, Wiesbaden, Germany) with DMEM/F12 (Gibco, Grand Island NY, USA) and FBS 10% (Gibco, Grand Island NY, USA) and incubated at 37°C with 5% CO^2^. The medium was replaced every 2 days. A tiny fraction of cells was collected and counted by Trypan Blue (Merck KGaA, Darmstadt, Germany). After five days, a colony with the typical spindle-like shape started forming, and at 80–90% of confluence, cells were harvested by enzymatic digestion by using a combination of Trypsin-EDTA (Gibco, Grand Island NY, USA) at 37°C for 5 minutes; suspended cells were collected and washed by PBS and centrifuged for 5 min at 3,000 rpm; the sediment of attached pellet at the bottom was removed by using serum-free media (Gibco, Grand Island NY, USA) and cultured in T-25 cm^2^ flask with 5 ml of DMEM/F12 with 10% FBS. The average amount of adherent cells was 0.9 (±0.37) × 10^6^ − 10^7^ cells per cm^2^. Cell viability at the time of passage was nearly 100%. The MSCs were able to proliferate up to 3^rd^ generation. The experiment was conducted by proceeding with same methodology with several fat tissue samples. Cells were tested by flow cytometry for MSC-specific cluster of differentiation (CD) markers to confirm their mesenchymal phenotype. The cells resulted negative for hematopoietic cell markers including CD14, CD45, and HLA-DR and were positive for exclusive markers such as CD73, CD90, and CD105.

### 2.2. Differentiation of AT-MSCs to Chondrocytes In Vitro

The MSCs at 80 to 90% of confluence were collected and transferred in an appropriate container and induced to chondrogenic phenotype by using a chondrogenic differentiation kit (StemPro™ Chondrogenesis Differentiation Kit, Thermo Fisher, Foster City, CA, USA), following the indications of manufacturer's instructions. MSCs were collected and washed to eliminated basal medium culture and were eventually cultured with the chondrogenic medium seeded on derm collagen scaffold and incubated at 37°C for a period between 2 and 3 weeks; the medium was changed every 3 days. Chondrocyte-like cells were then stained by Alcian Blue, Safranin-O, and hematoxylin and eosin (H-E) stain [16]. All stained samples were observed at a Nikon Eclipse 1000 optical microscope with different magnifications (Nikon Corporation, Tokyo, Japan).

### 2.3. Alcian Blue Staining Procedure

Alcian Blue (Merck KGaA, Darmstadt, Germany) is a dye used to assess the presence of chondrocytes; the stain reveals the sulphated proteoglycan in cartilage tissue [16]. Cell samples were deparaffinised with xylene substitute, followed by 3 changes of 5 minutes per change. Then, each sample was hydrated in 100% ethanol solution followed by 2 changes of 2 minutes each. The 3^rd^ step was the hydration of samples in 95% ethanol followed by 2 changes of 2 minutes each and again a hydration in 70% ethanol for 2 minutes with a subsequent hydration in 50% ethanol for 2 minutes. At this step, samples were rinsed in di-H_2_O for 5 minutes and incubated in 3% acetic acid for 3 minutes and subsequently stained with 1% Alcian Blue solution with a pH 2.5 for 30-60 minutes washed with tap water for 2 minutes and rinsed in di-H_2_O. The samples were cleared in xylene substitute with 2 changes of 2 minutes each.

### 2.4. Safranin-O Staining Procedure

Safranin-O (Merck KGaA, Darmstadt, Germany) allowed to confirm the presence of active chondrocytes by detecting the presence of cartilaginous proteoglycans, mucin, and mast cell granules on a formalin-fixed, paraffin-embedded cartilage tissue-like sample [16]. The final stain confirmed the presence of cartilage-like tissue and mucin in orange/red color, cell nuclei were stained in black, and the background appeared in bluish-green stain. Cell samples were deparaffinised, placed on a slide rinsed with distilled water, stained for 10 minutes with Weigert's iron hematoxylin, and washed with running tap water for 10 minutes. The cells were then stained with fast green solution for 5 minutes, rinsed with acetic acid solution, and stained in 0.1% solution of Safranin-O for 5 minutes. The samples were dehydrated and cleared with ethyl, alcohol (95%), ethyl alcohol (100%), and xylene for 2 minutes each, and resinous medium was used to fix.

### 2.5. The H-E Staining

The H-E staining technique is one of the principal tissue stains used in histology. The hematoxylin stains cell nuclei blue, and eosin stains the extracellular matrix and cytoplasm pink, with other structures taking on different shades, hues, and combinations of these colors. H-E stain was performed using a diagnostic kit (Celnovte Biotech Zhengzhou City, Henan Province, China). Moreover, in this study, the H-E Saigon (H-SG) staining method was presented (under patenting). This type of unique stain is similar to the more conventional H-E staining methodology, and the procedure however is highly sensitive for type 2 collagen and connective tissues. The samples were incubated in Bouin's solution to bind the dye to the tissue and let overnight at room temperature. Then, the samples were briefly rinsed under water thoroughly and with Weigert's iron hematoxylin and phosphotungstic acid (PTAH) for 10-15 minutes then rinsed under water for 5 minutes. The successive step uses Orange-G to stain the sample for 5-10 minutes, followed by rinsing under water for 2-3 minutes. Then, it is possible to proceed with one step blue/green (blue methylene/fast green) for 10-15 minutes and straight to fuchsin acid 1%, for 1 minute. Finally, the sample was rinsed under running water, dehydrated, cleared, and mounted in a diagnostic slide (all reagents were purchased from Merck KGaA, Darmstadt, Germany).

### 2.6. RT-PCR Procedure

The chondrocyte phenotype obtained from AT-MSCs was evaluated for the expression of chondrocyte-/cartilage-specific markers, the Cartilage Oligomeric Matrix Protein (COMP), collagen X, alpha-1 polypeptide (COL10A1), and the Collagen II, Human Tagged ORF Clone (COL2A1) by using the reverse transcription polymerase chain reaction (RT-PCR) following the manufacturer's instructions. RNA was isolated by using TRIzol (Gibco, Grand Island NY, USA) following the manufacturer's protocol. The extracted RNA was dissolved in diethylpyrocarbonate-treated water and successively transcribed to cDNA by using RT-PCR. cDNA has been amplified by using Applied Biosystems ABI GeneAmp PCR (Thermo Fisher, Foster City, CA, USA) system at 94°C for 40 s, 56°C for 50 s, and 72°C for 60 s for 35 cycles after an initial denaturation at 94°C for 5 min [15, 17, 18], using the primers reported in Table 1 and actin as internal control.

### 2.7. Collagen Matrix Scaffold from Skin Samples

A collagen matrix scaffold was obtained from human skin samples donated by consent patients from the Traumatic Unit of the Ho Chi Minh City Orthopaedics Traumatic Hospital. The samples were collected with previous official permission of three independent institutions: (I) Orthopaedic Traumatic Hospital Ethic Committee; (II) Ho Chi Minh City Health Department Ethic Committee; and (III) Ministry of Health of Socialist Republic of Vietnam (MOH).

Skin sample of the size of 3 cm × 3 cm was collected in appropriate containers with sterile buffer PBS solution with gentamycin and brought to the laboratory facility. The samples were processed in a sterile environment and cleaned from blood vessels, adipose tissues, and debris preserving only the dermis and epidermis. Cleared samples were successively treated with NaCl 1 M, EDTA, hypotonic solution, SDS solution, and PBS.

After each step with the above solution, the samples were collected and centrifuged at room temperature at 200/rpm for 5 minutes for 3 times; the samples were then collected and put in cold temperature to dry up. This step involved 3 subphases in a refrigerator at 4°C for 30 minutes, at -20°C for 1 hour and, eventually moved at -80°C for 24 hours. The samples were successively stored for 48 hours at 0.4 millibar pressure. Samples were packed and sterilized with gamma rays at a dose of 25 kGy and stored at 4°C.

Collagen matrix was evaluated before and after by two specific methods, the gel permeation chromatography (GPC) and the high-performance liquid chromatography (HPLC) to quantify collagen content in collagen dermal scaffold before and after treatment to monitor changes in collagen content in the steps of the treatment process. Scaffold samples with and without chondrocytes were then photographed at an APREO scanning electron microscope (SEM) (Thermo Fisher, Foster City, CA, USA).

In addition, a test of toxicity was performed to assess a grade of negative effect on cells by the collagen scaffold (ISO: 10993-5), at 24 hours, 48 hours, and 1 week to show that all samples were eventually free of toxins or any other dangerous alteration, and histology outcomes confirmed the good growth and features of implanted cells.

### 2.8. Flow Cytometry Analysis and Procedure

The AT-MSCs, once reaching the enough confluence (80-100%), were enzymatically harvested by Trypsin-EDTA (0.25%-0.02%). The cell suspension was centrifuged at 3000 rpm for 5 minutes. Pellets were then washed two times by PBS and analysed by flow cytometry for CD panel expression: CD45, CD34, CD11b, CD13, CD19, CD14, CD105, CD73, and CD90. The analysis was performed 3 times, (modality 2-laser, 6-color) by BD FACSCanto™ (Amersham Biosciences Corp, Piscataway, NJ, USA), (2-laser, 6-color).

### 2.9. The Construction of Cartilage-Like Membrane by Using Human AT-MSC Chondrocytes

At the 3^rd^ passage, the AT-MSCs were induced to chondrocytes by using the StemPro™ Chondrogenesis Differentiation Kit. Chondrocytes at 90% confluence were enzymatically harvested, 10^6^–10^7^ cells/cm^2^, and seeded on the prepared skin collagen scaffold by using the centrifugation force of 1000 rpm for 1 minute and repeated 5 times. Cells and scaffold were successively stained by H-E and Safranin-O and analysed by SEM to assess a 3D presence and distribution of cells on the scaffold.

## 3. Results

### 3.1. Isolation Culture, Differentiation of AT-MSCs, and Their CD Marker Expression

After being isolated and collected from adipose tissues, cells were successively immerged and cultured into a basal DMEM plus 10% FBS. The CD panel expression is as follows: CD13, CD105, CD73, and CD90.

**Figure 2:**
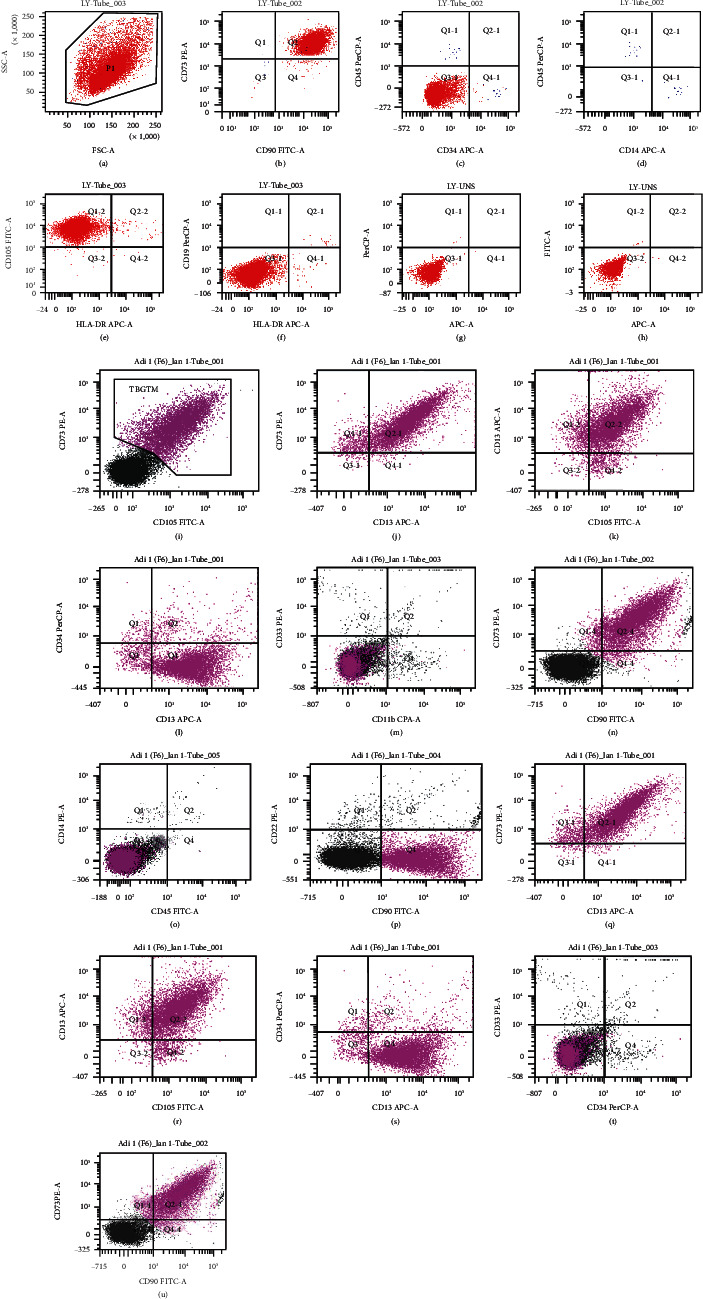
Flow cytometry results confirmed the presence of MSC markers (a–u) CD13^+^CD73^+^CD90^+^CD105^+^ and SSC^low^ and a negative expression for CD 11b, CD14, CD19, CD34, CD45, and HLA-DR.

Flow cytometry results showed positive expression for CD13^+^CD73^+^CD90^+^CD105^+^and SSC^low^ and negative expression for markers CD34, CD11b, CD14, CD19, CD45, and HLA-DR (APC-A (allophycocyanin); fluorescein isothiocyanate (FITC); forward scattered area (FSC-a); cytotoxic cryopreservant (Per-CPA)) (Figure 2).

### 3.2. AT-MSC-Derived Chondrocytes In Vitro Stains and RT-PCR Outcomes

**Figure 3:**
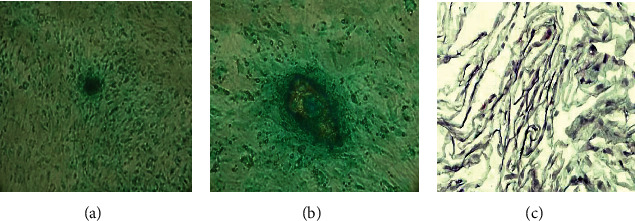
AT-MSC chondrocyte-like cells at 14 days: (a) Alcian Blue magnification ×10; (b) Alcian Blue magnification ×40; (c) H-E stain of chondrocytes magnification ×20.

At week 2, the AT-MSCs were collected and induced to chondrocytes by using the StemPro™ Chondrogenesis Differentiation Kit. The chondrocytes were successively stained by H-E and Alcian Blue. After 14 days, the result was a typical cluster of spindle-fibroblast-like cells which were largely present (Figure 3).

**Figure 4:**
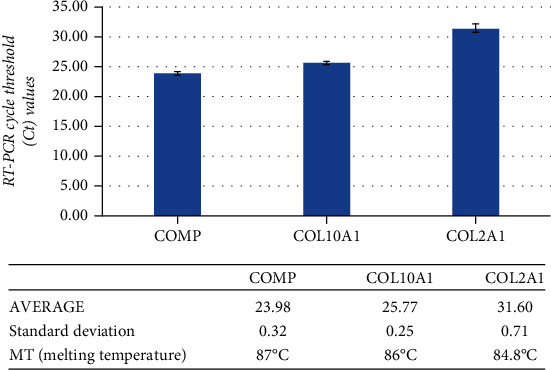
RT-PCR cycle threshold shows a positive expression for cartilage extracellular matrix genes: (a) the Cartilage Oligomeric Matrix Protein-COMP; (b) collagen type 1-COL10A1; (c) the collagen type 2-COL2A1.

The RT-PCR analysis procedure was used to assess the expression of typical cartilage extracellular matrix genes like the COMP, COL10A1, and COL2A1, showing reverse transcription. Genes have been detected with a cycle threshold (Ct) value of less than 38 cycles of amplification (Figure 4). Is well known that cycle threshold (Ct) values inversely correlate to the amount of target nucleic acid in the sample. According to universal standard protocols, a positive result indicates that investigated markers were detected with a Ct value of less than 38 cycles of amplification.

### 3.3. Results of Collagen Scaffold Obtained from Human Derm-Skin

**Figure 5:**
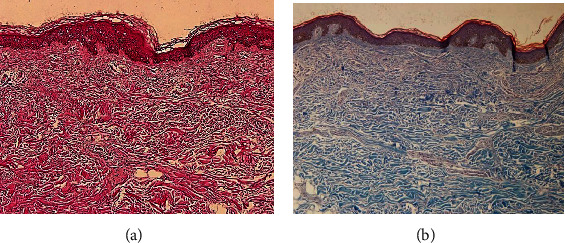
Human skin before the process of creating the skin-derm scaffold at magnification 10x: (a) H-E stain; (b) H-SG stain.

**Figure 6:**
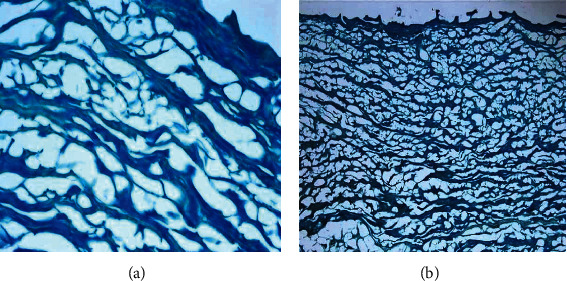
Human-derived derm-skin after removal of the epidermis, fat tissues, blood vessels, and debris part: (a) H-SG stain, magnification 60x; (b) H-SG stain magnification 10x.

**Figure 7:**
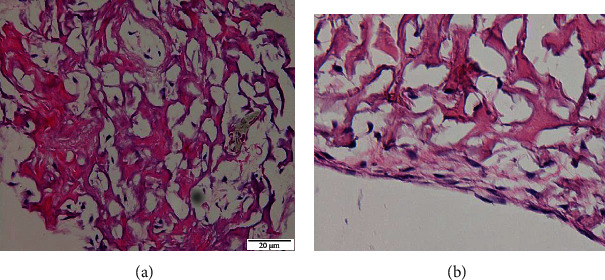
Human-derived derm-skin cartilage with attached mature chondrocytes at 21 days: H-E stain, magnification 20x (a) and magnification 40x (b).

**Figure 8:**
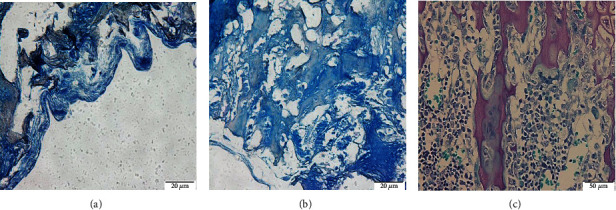
Cartilage-like tissue stained by Alcian Blue: magnification 20x (a), 40x (b), and chondrocytes embedded in cartilage matrix Alcian Blue at day 21 (magnification 50x) (c).

**Figure 9:**
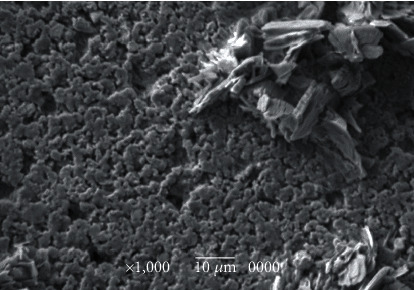
Derm-skin scaffold with collagen sheet, without cells, at SEM magnification ×1,000 (bar: 10 *μ*m). The scaffold proved to have adequate intercompartmental space to allot and home chondrocytes to build up a final cartilage-like matrix.

**Figure 10:**
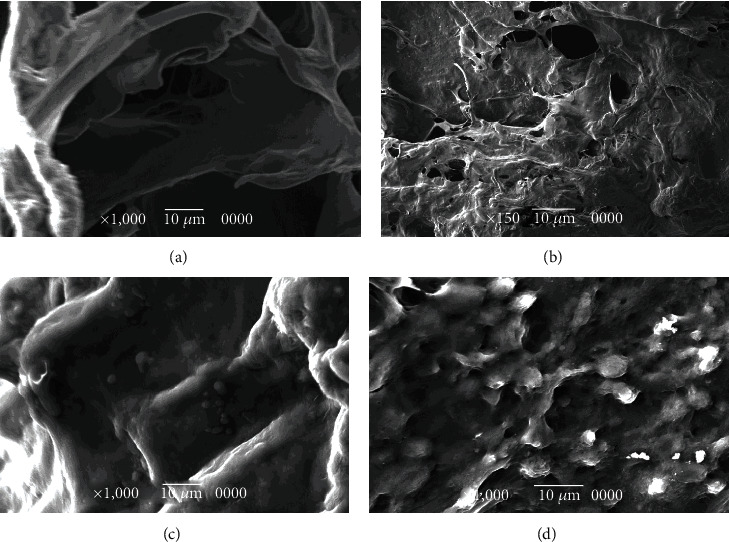
(a, b) Derm-skin scaffold with collagen sheet, without chondrocytes at SEM magnification ×1,000 (a) and magnification ×150 (b). (c, d) The SEM shows the presence of well-integrated chondrocytes at 15-21 days, magnification ×1,000. The chondrocytes were able to construct and modify the scaffold environment.

The derm-skin collagen matrix scaffolds, obtained from human skin samples and then deeply cleaned and enzymatically treated to obtain a pure matrix without cells or residuals such as blood, fat deposits, or debris, followed a final sterilization with gamma irradiation. The untreated skin samples were stained by H-E and H-SG (Figures 5–8), and the obtained scaffolds were analysed using SEM. The derm/skin scaffolds showed to have enough intercompartmental space to allocate and home chondrocytes to build up a final cartilage-like matrix (Figures 9 and 10).

## 4. Discussion

In this translational study, we chose to use human dermal substrate as vector to transduce human AT-MSC-derived chondrocytes since the human skin/dermis remains one of the most efficient sites of type II collagen matrix, significant to accommodate chondrocytes in generating very close biosimilar cartilage-like tissue [19, 20]. After a long-term follow-up (11 years) on 61 patients after autologous chondrocyte transplantation graft procedures, it was observed that all of the specimens stained positive for COMP and aggrecan. Hyaline-like cartilage stained positive for type II collagen in the predominant part of the tissue (>50%). The fibrous area stained positive for type I collagen stiffness measurements in hyaline cartilage which were twice those in fibrous cartilage samples [21]. Basically, the repair mechanism was seen in lesions that were fibrous in appearance, and patients received native type II collagen (with or without drug support like acetaminophen) which showed good to excellent long-term outcomes [22, 23].

Therefore, we anticipate the use of skin/derm as a high functional biovector for delivery of substitute cartilage-like substrate. Such approach would potentially allow surgeons to treat articular cartilage lesions with transduced MSCs in a comfortable level of security and in a relatively short healing period.

Furthermore, this strategy may allow us to avoid the current adversities coming from the two step-operative methodology of current cartilage regenerative approach that involves the choice of compatible biomaterial, the generation of *in vitro* chondrocytes from MSCs, and their insertion into the damaged articulation. The *in vitro*-derived MSC chondrocytes located within fibrous injured tissue are primarily spindle-shaped fibroblast-like cells capable of synthesizing type I collagen that is not fully compatible with the *in vivo* normal articular chondrocyte/cartilage tissue conformation. The outcomes of this technique showed the formation of fibrocartilaginous or bone-like tissues both histologically and mechanically different from the surrounding endogenous cartilage tissue [21–24]. These types of side effects are also common even with the use of the latest generation of biomaterial solutions such as fibrin glue, acellular matrix, collagen gels, and alginate up to hyaluronic acid-derived oligostilbenoids, polylactic acid, and polyglycolic acid [25–32].

Technically, the use of autologous chondrocytes is not always a practice free of several issues. Indeed, they must be expanded *in vitro* before being implanted during a second surgery that is not always an easy practicability; on the other hand, the use of autologous cartilage is strictly reserved to the short availability of autologous tissue.

Thus, the limitations are seen either in terms of biointegration or purely as surgical procedure. For instance, fibrin glue inserted might be gradually replaced by fibrous tissue in a period of less than 21 days and it could function as the main barrier against chondrocyte migration which constrains the correct healing process [20, 33]. The typology of chondrocytes obtained are collagen type I producer and a two weeks' analysis and confirmed a sensitive low and less amounts of both cartilage-specific chondrocyte DNA and proteoglycans compared to those seeded with type II collagen sponges [34, 35]. With regard to hyaluronic compounds, though representing a valid support in cartilage regenerative therapy, these products still remain too fluid to be used as functional scaffolds to accommodate cells. In addition, this procedure does not solve the problem of collagen type II matrix, essential for a full recovery of functional cartilage tissue. Moreover, the structural architecture of the scaffold is equally important. Besides, it should be mentioned that quite limited other factors may compromise a correct growth and development of chondrocytes toward their use in cartilage repair and, as studies have investigated, basal medium used to growth cells *in vitro* may also eventually negatively affect this process [36].

In addition, vitamin D appears to play an important role in bone and cartilage metabolism since its receptors are widely found in human articular chondrocytes. Thus, effects of variation of vitamin D and its supplementation in diet may directly impact cartilage and bone biology [37–40].

Some studies suggested the direct use of seeded chondrocytes on specific semisolid elastic biomaterials that exhibited the capacity of these cells to produce cartilage *in vivo*, due to intrinsic inductive mechanism of the defected cartilage that may act as a “bioactive chamber with natural settings” [40–46].

Therefore, those findings, in line with our results, showed that it is actually possible to carry on with one-step graft and implantation surgery procedure using a very ductile biocompatible material like the autologous skin/derm. This option may improve the quality of the regenerated articular cartilage by supporting cell adhesion, cell growth, and gene expression, specific for cartilage regeneration. Our findings showed that the dermal collagen may exert important effects on the quality of *in vitro* expanded chondrocytes, leading in this way that the influence of collagen skin matrix helps to produce highly active and functional chondrocytes for long-term cartilage tissue regeneration. The presented data showed that skin/derm can function as a very reliable bioscaffold for AT-MSC-derived chondrocytes and we can also speculate that the obtained scaffold fit into an articular cartilage defect (Figures 5–8). Chondrocytes seeded onto derm/skin scaffold acquired the typical columnar conformation and shape of those found on human cartilage (Figures 9 and 10). This solution avoids also collateral effects and rejection associated with allograft transplants. Thus, the idea was to combine a very ductile technical solution with autologous MSC transplantation that not necessarily should come from adipose tissue but from the bone marrow, dental compartment, synovial fluid, and/or peripheral blood.

## 5. Conclusion

In summary, there is a huge promise to advance current cartilage therapies toward achieving a consistently successful approach for addressing cartilage afflictions. The results of the current *in vitro* study showed a biocartilage graft solution, which demonstrated chondrocyte formation. These findings confirm that the cells on the skin/derm scaffold were able to produce cartilage tissue gradually throughout collagen type I and II matrix as well as on the surface of the substrate. Therefore, these results may be considered a valid and solid first step toward the generation of a complete and full-scale compatible portion of cartilage-like tissue containing both chondrocyte and essential substrates such as collagen type II and solid cartilage to support and replace injured tissues. Further *in vivo* research and investigation are necessary to establish whether this approach would be pertinent in the context of articular joint cartilage repair. Translational cooperation between research groups and clinicians is key to the safe and successful progression of tissue engineering for cartilage regeneration therapy, with the ultimate goal of providing all patients who have cartilage-related diseases with the opportunity to repair and conserve their joint rather than replacing it.

## Figures and Tables

**Table 1:** RT-PCR primers used to identify, respectively, COMP, COL10A1, and COL2A1 genes.

*NM_000095* (*GenBank*) COMP (Cartilage Oligomeric Matrix Protein)	*NM_016685.2* (*GenBank*) *Mus musculus cartilage oligomeric matrix protein (COMP)*	*NM_000493* (*GenBank*) COL10A1 (collagen X, alpha-1 polypeptide)	*NM_001844* (*GenBank*) COL2A1 (Collagen II, Human Tagged ORF Clone)
Forward GCTCTGTGGCATACAGGAGA Reverse CATAGAATCGCACCCTGATG	Forward TGGGTGATGCCTGTGATAGT Reverse CGTCATTGTCATCATCGTCA	Forward CGCTGAACGATACCAAATGCCC Reverse TGGACCAGGAGTACCTTGCTCT	Forward CCTGGCAAAGATGGTGAGACA Reverse CCTGGTTTTCCACCTTCACCTG

## Data Availability

The experimental data used to support the findings of this study are included within the article.
